# The value of intraoperative MRI for resection of functional pituitary adenomas—a critical assessment of a consecutive single-center series of 114 cases

**DOI:** 10.1007/s10143-022-01810-7

**Published:** 2022-05-14

**Authors:** Moritz Scherer, Paul Zerweck, Daniela Becker, Lars Kihm, Jessica Jesser, Christopher Beynon, Andreas Unterberg

**Affiliations:** 1 Department of Neurosurgery, Heidelberg University Hospital, Im Neuenheimer Feld 400, 69120 Heidelberg, Germany; 2 Department of Endocrinology, Heidelberg University Hospital, Heidelberg, Germany; 3 Department of Neuroradiology, Heidelberg University Hospital, Heidelberg, Germany

**Keywords:** Functional pituitary adenoma, Transsphenoidal surgery, Intraoperative MRI, M. Cushing, Hypercortisolism, Prolactinoma, Diabetes insipidus

## Abstract

This series sought to evaluate the role of intraoperative MRI (iMRI) for resection of functional pituitary adenomas (FPAs). We retrospectively reviewed clinical data of 114 consecutive FPAs with excessive hormone secretion treated with transsphenoidal surgery and iMRI during 01/2010–12/2017. We focused on iMRI findings, extend of resection and postoperative hormonal remission. Variables of incomplete resections and persistent hormone excess were evaluated by binary regression. Patients with FPAs presented with hypercortisolism (*n* = 23, 20%), acromegaly (*n* = 56, 49%), and as prolactinomas (*n* = 35, 31%) resistant to medical treatment. Preoperative MRI showed 81 macroadenomas (71%) and optic system involvement in 41 cases (36%). IMRI was suggestive for residual tumor in 51 cases (45%). Re-inspection of the cavity cleared equivocal findings in 16 cases (14%). Additional tumor was removed in 22 cases (19%). Complete resection was achieved in 95 cases (83%). Postoperative morbidity was low (1.7% revision surgeries, 0.8% permanent diabetes insipidus). Overall hormonal remission-rate was 59% (hypercortisolism 78%, acromegaly 52%, prolactinoma 57%). Supra- and parasellar invasion and preoperative visual impairment were significant predictors for incomplete resections despite use of iMRI. Risk for persistent hormone excess was increased sevenfold after incomplete resections. IMRI enabled reliable identification of tumor remnants during surgery and triggered further resection in a considerable proportion of cases. Nevertheless, tumor size and invasiveness set persistent boundaries to the completeness of resections. The low rate of surgical complications could point at a less invasive iMRI-guided surgical approach while achieving a complete tumor resection was a crucial determinant for hormonal outcome.

## Introduction

Functional pituitary adenomas (FPAs) represent a unique subentity among pituitary adenomastumors of the sellar region. They account for approximately 1/2 to 2/3 of pituitary adenomas and most frequently they affect the lactational (32–66%), growth hormone (8–16%), and adrenocorticotrope (2–6%) hormone axes[1].

Surgical tumor resection has been established as a cornerstone for Cushing’s disease and acromegaly, while it is regarded second-line treatment after medical treatment with dopamine agonists in prolactinomas[2–4]. Transsphenoidal resection is generally the approach of choice while microscopic and endoscopic resection techniques are currently well established with comparable results regarding efficacy and safety[5].

Besides leading to decompression of surrounding nerve structures such as the optic system, the extent of resection (EOR) and gross resection of FPAs have been shown to strongly correlate with outcome and enable remission of hormone excess and normalization of pituitary function[2–4]. However, radical tumor resection may also increase the risk of pituitary gland insufficiency following invasive manipulation of pituitary gland tissue. Since its introduction into neurosurgery, intraoperative MRI (iMRI) has been increasingly used as a surgical adjunct for improvement of intraoperative identification of residual tumor and to enhance the rates of complete tumor resections in pituitary surgery with and without endocrine symptoms [6–10]. In this regard, iMRI potentially exhibits a high value for resection guidance and quality control especially in FPA, as surgical treatment of these tumors can be challenging.

Previous medical therapy in prolactinoma can lead to fibrous tissue alterations of the adenoma impairing resection and delineation of gland tissue within the sella[3, 11]. For growth hormone–releasing tumors, acromegaly implies a high disease burden and adjuvant treatment with somatostatin analogues has been shown to severely impact the quality of life in cases where surgical treatment fails to control excess hormone levels[12]. Hypercortisolism is frequently caused by small microadenomas that are difficult to locate during surgery. IMRI can improve identification of adenomas in selective adenomectomy supporting the preservation of pituitary function[13].

In this series, we sought to review the value of iMRI for resection of FPAs with active hormone secretion emphasizing intraoperative findings, the extent of resection and postoperative hormone function in a consecutive single-center retrospective cohort of 114 cases.

## Patients and methods

### Patient selection

The institutional review board approved this retrospective study and the requirement for patient informed consent was waived (S-119/2018).

For this retrospective series, we identified consecutive cases of FPA with confirmed excess hormone secretion treated with transsphenoidal surgery (TSS) from a single academic institution from 01/2010 to 12/2017. Primary and repeat surgeries were included.

**Fig. 1:**
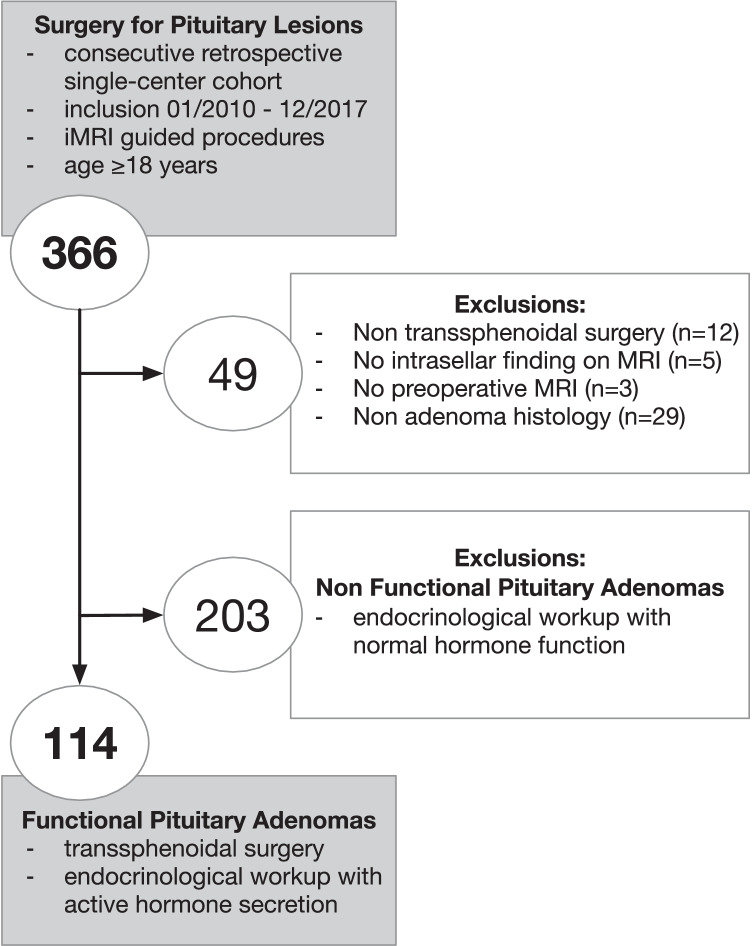
Flowchart of patient inclusion. Consecutive retrospective series of functional pituitary adenomas with active hormone secretion

Further inclusion criteria were age > 18 years, intrasellar pathology on preoperative MRI, and histopathological findings confirming pituitary adenoma. All surgeries were routinely performed with adjunct use of iMRI. Patients were excluded for non-transsphenoidal approaches, absence of intrasellar pathology on MRI, and any non-adenoma histology (see Fig. 1 for a flowchart of patient selection).

### Clinical parameters and endocrinologic evaluation

Clinical parameters extracted from patient records included age, sex, preoperative neurologic deficits, visual impairments, previous pituitary surgery, and previous medical or radiation therapy for a pituitary adenoma.

Endocrinologic verification of excess hormone production was mandatory for patient inclusion.

Diagnosis of ACTH-dependent Cushing’s syndrome with hypercortisolism was based on clinical presentation in combination with at least two abnormal diagnostic test results. Tests included morning serum cortisol, 24-h urine free cortisol, dexamethasone suppression test, plasma adrenocorticotrophic hormone (ACTH) levels, corticotropin-releasing hormone (CRH) stimulation test, or inferior petrosal sinus sampling in equivocal cases[14].

Growth hormone secretion and acromegaly were diagnosed from clinical findings, elevated Insulin-like growth factor (IGF-1) levels, and insufficient suppression of growth hormone (GH) after oral glucose tolerance test (OGTT)[15].

Hyperprolactinemia and prolactinoma were diagnosed by elevated prolactin serum levels (> 250 ng/ml) and clinical findings (e.g., amenorrhea, hyperlactation, sexual inappetence). Gonadotrope function was evaluated direct hormone testing[16].

All patients were treated with 100 mg of i.v. hydrocortisone on the day of surgery followed by an oral maintenance dose of 25 mg until testing of pituitary function 4–6 weeks after surgery.

Hormonal remission from hypercortisolism and Cushing’s disease was defined according to a consensus statement by morning serum cortisol < 5 µg/dl (< 140 mmol/liter)[14]. For acromegaly, remission was diagnosed with normalization of IGF-1 levels and GH levels < 1 ng/ml after OGTT[15, 17]. For prolactinoma, repeated serum levels < 25 ng/ml were required for remission[16]. Hormonal remission was recorded at the earliest 6 weeks after surgery or at any time it occurred during follow-up. For this study, hormonal remission was recorded, only when the patient was off any anti-hormonal medication.

### Image analysis

All patients had pre- and postoperative 1.5 or 3 T MRI. Additionally, 1.5 T intraoperative MRI was performed in all cases in this series applying isotrope sequences (1mm^3^ voxel size). Tumor extension was measured on T1w and T2w sequences with and without contrast manually on axial, coronal, and sagittal planes (in millimeters) and tumor invasion was evaluated according to the Knosp grade. Adenomas ≥ 10 mm were regarded macroadenomas. Image analysis was performed in conjunction by two experienced neurosurgeons (M.S. and C.B.) in this study. Postoperative extent of resection was recorded according to neuroradiology reports 8–12 weeks after surgery.

### Operative procedures and intraoperative MRI

All transsphenoidal resections were performed with the use of conventional microscopic surgery. At the discretion of the surgeon, an endoscope (0° or 30° rigid endoscope, Storz, Tuttlingen, Germany) was used for additional visualization of the cavity (i.e., endoscope-assisted resections). Endoscope-only procedures were not performed in this study. Neuronavigation was applied on an individual basis in patients with altered anatomy or repeated surgery.

**Fig. 2:**
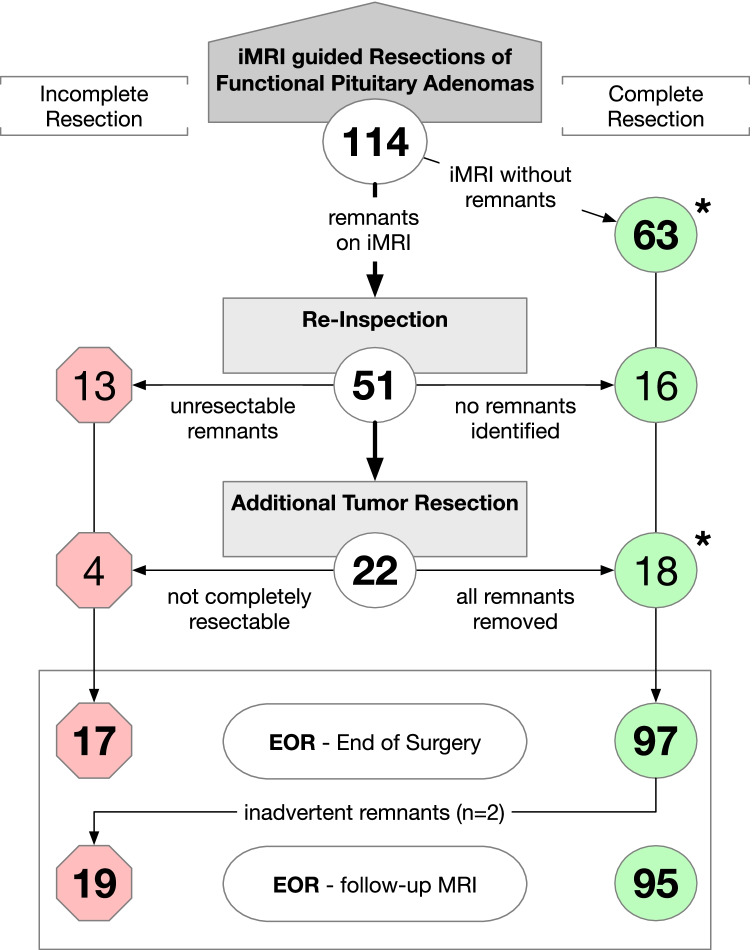
Flowchart of iMRI-guided resections of functional pituitary adenomas. During surgery, a re-inspection or additional tumor resection was performed to achieve either complete resections (case numbers in green circles on the right) or incomplete resections (case numbers in red octagons on the left). Extent of resection (EOR) was evaluated after surgery and according to follow-up MRI. In two cases, residual tumor was inadvertently found on follow-up MRI, despite a complete resection was assumed according to iMRI (false-negative iMRI findings). These cases are indicated by asterisks (*), respectively

**Fig. 3:**
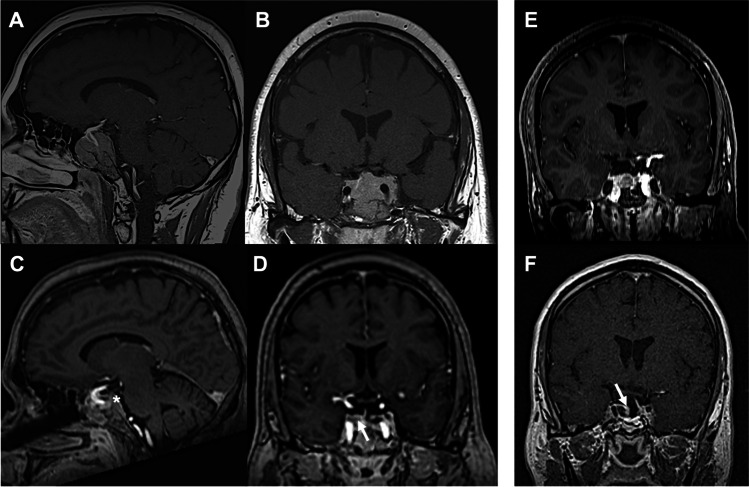
Illustrative cases (all T1w + CE). Case 1: Preoperative MRI of a large invasive adenoma with M. Cushing (**A** + **B**). Corresponding intraoperative MRI shows irresectable tumor remnants extending behind the clivus (**C**, highlighted by asterisk*) as well as intrasellar remnants removed by *additional resection* (highlighted by solid arrow in **D**). Case 2: Preoperative MRI in a case with acromegaly and a recurrent right-sided intrasellar tumor (**E**). On iMRI, residual adenoma was suspected associated to an intrasellar membrane (solid arrow in **F**). Upon *re-inspection*, no remnant could be identified however, which cleared this equivocal finding

All procedures were performed with the adjunct of intraoperative MRI (Siemens Magnetom Espree 1,5 T, Siemens, Erlangen, Germany) following a standardized protocol: After initial resection, iMRI was performed upon the surgeon’s request and was evaluated for tumor remnants in collaboration by the surgeon and attending neuroradiologist yielding a joined report. Subsequently, surgery could be either *terminated* (if no remnants were observed or if remnants were not accessible for further resection) or a *re-inspection* of the resection cavity could be performed (to clear equivocal findings) eventually followed by an *additional resection* (to remove further adenoma tissue) if possible (see Fig. 2). For reasons of time efficacy, only one iMRI scan was performed per surgical procedure. Figure 3 gives illustrative cases for intraoperative decisions for *additional resection* (A–D) and *re-inspection* (E–F).

### Statistical analysis

Continuous measures were stated in means with standard deviations. Categorical variables were given in frequencies and percentages of the whole cohort. The Mann–Whitney *U*-test was used to compare continuous variables of nonparametric data. Chi-square tests were used for contingency analysis of categorical variables. Variables of incomplete resections or persistent hormone excess were evaluated by univariate binary logistic regression analysis. Time to progression was determined according to imaging during follow-up and evaluated by the Kaplan–Meier estimates. Statistical analysis was performed by SPSS v24 and Graph Pad Prism v8. *p*-values < 0.05 were regarded as statistically significant.

## Results

### Study cohort and demographics

Initially, 366 consecutive surgeries for pituitary lesions were retrospectively identified. After exclusion of cases with non-transsphenoidal surgery (*n* = 12), non-functioning pituitary adenomas (*n* = 203), pathology other than pituitary adenoma (*n* = 29), missing preoperative imaging (*n* = 3), and absence of intrasellar findings (*n* = 5), 114 FPAs were included for further analysis in this study (see Fig. 1).

Within this cohort of FPAs, there was a 60% predominance for females. Median age was 42.5 years at the time of surgery (range 18–74 years). Follow-up with full endocrinologic evaluation was available for 103 patients (90%). Median follow-up was 8 months (range 1–97 months, interquartile range 4–22 months). Three tumor progressions were recorded during follow-up. Three cases received additional transcranial surgery for removal of tumor remnants.

### Clinical manifestations

All 114 cases in this study had FPAs with endocrinologically confirmed excess hormone secretion showing ACTH-dependent hypercortisolism (Cushing’s disease) in 23 cases (20%), growth hormone–related acromegaly in 56 cases (49%), and hyperprolactinemia caused by prolactinoma in 35 cases (31%), respectively.

Clinical signs of hormone excess were observed in 93 cases (82%) mostly affecting acromegaly (e.g., growth of nose, ears, or hands, *n* = 45) and Cushing’s disease patients (e.g., obesity, striae, *n* = 23). Secondary amenorrhea was the leading clinical symptom in 8 patients with prolactinoma. Seven patients suffered from hyperlactation. Visual disturbances were reported by 14 patients in this study (12%). Eleven patients had received previous pituitary surgery for Cushing’s disease (*n* = 2), acromegaly (*n* = 7), and prolactinoma (*n* = 2). All prolactinoma patients had received medical treatment prior to surgery (*n* = 35) but showed dose intolerance to dopamine analogues (*n* = 8, 23%) or insufficient treatment effects (*n* = 22, 63%) leading to surgery. Four patients (11%) requested surgery in addition to medical treatment and one patient (3%) was surgically treated for new rhinoliquorrhea following treatment with dopamine analogues.

Additional partial pituitary insufficiency was diagnosed in 20 cases (18%) mostly associated with prolactinoma (*n* = 11, 55%) followed by the gonadotropic and thyrotropic axes.

### Imaging manifestations

Preoperative MRI showed intrasellar microadenomas in 33 cases and macroadenomas in 81 (71%). Mean tumor diameters were 14 ± 8 mm, 14 ± 9 mm, and 15 ± 10 mm on coronal, sagittal, and axial planes, respectively. Invasive growth pattern was assessed by Knosp grade (Table 1) and showed extensive invasion into the cavernous sinus (Knosp grade 3 + 4) in 22 cases (20%). Pure intrasellar lesions (Knosp grade 0 + 1) were found in the majority of cases (*n* = 78, 68%). Suprasellar extension of tumors with contact and dislocation of the optic chiasm was observed in 41 cases (36%). The sphenoid sinus was infiltrated by 11 adenomas (10%). Microadenomas were significantly more frequent in Cushing’s disease (16/23, 70%) compared to acromegaly (11/56, 20%) or prolactinomas (6/35, 17%), *p* < 0.0001 (*X*^2^ test). A majority of adenomas showed contrast enhancement (*n* = 75, 66%). Intratumoral cysts were observed in 44 cases (39%).

**Table 1:** Demographics

		All cases (*n* = 114)	
		*N*	%
Sex (f/m)		70/44	61/39
Age (median, range)		42.5y	(18–74y)
Visual deficits		14	12
**Hormone-active adenomas**		114	100
**M. Cushing** (Hypercortisolism)		23	20
**Acromegaly** (Growth hormone secreting)		56	49
**Prolactinoma** (Hyperprolactinemia)		35	31
Previous pituitary surgery		11	10
Previous medical therapy		35^1^	31
Previous radiation therapy		0	0
Macroadenoma		81	71
Knosp grade	0	29	25
	1	49	43
	2	14	12
	3	9	8
	4	13	12
Sagittal size	< 10 mm	44	39
	10 < 20 mm	55	48
	20 < 30 mm	10	9
	> 30 mm	5	4
Contact with optic chiasm		41	36
Microscopic surgery		70	61
Endoscope assisted surgery		44	39
Full endoscopic surgery		0	0
Use of navigation		52	46

^1^All for hyperprolactinemia

### Transsphenoidal surgery with adjunct of iMRI

All surgeries were performed by either of three senior surgeons in this study and iMRI was used for intraoperative control of the extent of resection in all cases. Endoscopic visualization of the resection cavity was performed in 44 cases (30%). Neuronavigation was used in 52 (46%) of cases.

For an illustration of the iMRI workflow and resection results, see the flowchart in Fig. 2.

After an initial resection attempt, iMRI was performed to estimate the extent of resection. On iMRI, residual tumor was suspected in 51 cases (45%) while iMRI demonstrated complete resection in 63 cases (55%).

When iMRI was suggestive for remnants, a careful re-inspection of the resection cavity aided to rule out equivocal iMRI findings without detection of residual tumor in 16/114 cases (14%) (see Fig. 3E, F for an illustration). Following confirmation of a complete tumor resection on postoperative MRI, these 16 cases were regarded false-positive iMRI findings.

By re-inspection, identified residual tumor portions were not deemed amenable to further resection in another 13/114 cases (11%) and surgery was terminated after iMRI.

Among the 51 cases with suspected remnants on iMRI, less than half of those were eventually amenable to further tumor resection (22/114 cases, 19%) (see Fig. 3A–D). Further tumor resection led to a complete removal of remnants in 18 cases while minor remnants were intentionally not re-approached in 4 cases due to a non-acceptable risk of morbidity.

Following iMRI-guided surgery, a complete tumor removal was assumed in 97 patients (85%), while incomplete resection was achieved in 17 cases (15%).

Final extent of resection was evaluated according by postoperative MRI 8–12 weeks after surgery. A complete tumor resection was observed in 95 cases (83%) while residual tumor was detected in 19 cases (17%) (Table 2). This implicated false-negative intraoperative findings in 2 cases (1.8%). In one case with acromegaly, iMRI suggested a complete resection but small intrasellar remnants could be identified postoperatively. In the second case with prolactinoma, unanticipated remnants were found postoperatively despite an additional resection after iMRI and a presumed complete resection at the end of surgery.

**Table 2:** Extent of resection and hormonal outcome

		Complete tumor resection		Incomplete tumor resection			Hormonal remission		Persistent hormone excess		
	*n*	*n*	%	*n*	%	*p*-value	*n*	%	*n*	%	*p*-value
M. Cushing	23	20	(87)	3	(13)	0.63	18	(78)	4	(17)	0.17
Acromegaly	56	47	(84)	9	(16)		29	(52)	20	(36)	
Prolactinoma	35	28	(80)	7	(20)		20	(57)	12	(34)	
**Total**	**114**	**95**	**(83)**	**19**	**(17)**		**67**	**(59)**	**36**	**(32)**	
Missing follow-up							11	(10)			

Accordingly, our iMRI-based approach to FPA resections had a 94.6% (95% C.I. 82.3–99.0) sensitivity to detect tumor remnants and a 79.2% (95% C.I. 68.9–86.8) specificity. Positive predictive value of suspected tumor remnants on iMRI was 68.6% (95% C.I. 54.9–79.7), due to 16 false-positive findings. When iMRI did not show residual tumor, this was in agreement with postoperative MRI in 96.8% (95% C.I. 89.1–99.4) (negative predictive value).

### Variables of incomplete resections despite use of iMRI

We evaluated variables of incomplete resections by binary regression analysis (Table 3). Sagittal tumor size (HR 4.43 *p* < 0.0001) and parasellar invasion into the cavernous sinus graduated by the Knosp grade (HR 3.22, *p* < 0.0001) as well as contact with the optic chiasm (HR 3.77, *p* = 0.01) and preoperative visual deficits (HR 4.25, *p* = 0.02) indicated a higher risk for incomplete resections. Neither previous surgery nor medical therapy, FPA entity, or use of additional surgical tools like endoscopy or navigation had a significant impact upon the extent of resection in our series. This illustrates how tumor size and invasiveness set persistent boundaries to the completeness of a resection despite use of iMRI or other surgical tools.

**Table 3:** Univariate regression for variables of an incomplete resection in iMRI-guided surgery

	HR	95% C.I		*p*-value
		Lower	Upper	
Sex (f/m)	**3.25**	1.22	8.67	**0.02**
Age (cont.)	0.99	0.96	1.03	0.81
**Visual deficits** (y/n)	**4.25**	1.29	14.0	**0.02**
M. Cushing (y/n)	0.61	0.16	2.27	0.46
Acromegaly (y/n)	0.93	0.36	2.40	0.88
Prolactinoma (y/n)	1.50	0.56	4.04	0.42
Previous pituitary surgery (y/n)	1.24	0.31	4.91	0.76
Previous medical therapy (y/n)	1.50	0.56	4.04	0.42
**Knosp grade** (cont.)	**3.22**	2.03	5.10	** < 0.0001**
**Sagittal size** (cont.)	**4.43**	2.14	9.18	** < 0.0001**
**Contact with optic chiasm** (y/n)	**3.77**	1.41	10.11	**0.01**
Microscopic surgery only (y/n)	*0.39*	0.15	1.03	0.06
Use of navigation (y/n)	0.87	0.34	2.27	0.78

**Bold** indicating significance, *HR*: hazard ratio

Table 4 provides a detailed overview of cases in which resections remained incomplete despite iMRI guidance and re-inspection or additional resection. Moreover, intraoperative findings and the decisions whether to re-approach the tumor remnants are presented.

**Table 4:** List of cases with tumor remnants after iMRI-guided transsphenoidal surgery. Sagittal size is given in groups of < 10 mm, 10 < 20 mm, 20 < 30 mm, and > 30 mm. *ICA* internal carotid artery, *NA* not available, *PD* progressive disease, *SD* stable disease

	Entity	Knosp grade	Sagittal size (in mm)	Contact with optic chiasm	Previous pituitary surgery	Location of tumor remnant	Termination of surgery after	Reason for termination of surgery	Follow-up			Adjuvant treatment
									Hormones	Radiology	Duration (months)	
**1**	Acromegaly	3	> 30	Yes	No	Covering ICA	iMRI	Risk of morbidity	Persistent excess	SD	8	Repeat surgery @ 8 m with hormonal remission
**2**	Prolactinoma	4	10 < 20	Yes	No	Superior to optic chiasm	iMRI	Not reachable	Remission	SD	9	
**3**	Acromegaly	1	10 < 20	Yes	No	None	iMRI	False-negative iMRI finding	Persistent excess	PD	22	Somatostatin + radiation therapy
**4**	Acromegaly	4	20 < 30	Yes	No	Cavernous sinus	iMRI	Risk of morbidity	Remission	SD	8	
**5**	Prolactinoma	4	> 30	Yes	No	Cavernous sinus	iMRI	Risk of morbidity	Persistent excess	SD	8	Dopamin
**6**	Acromegaly	3	< 10	No	No	Cavernous sinus	iMRI	Risk of morbidity	Persistent excess	SD	7	Somatostatin
**7**	Prolactinoma	1	20 < 30	Yes	No	Intrasellar	iMRI	Dense tumor capsule	Remission	SD	76	
**8**	Prolactinoma	3	10 < 20	No	No	Cavernous sinus	iMRI	Risk of morbidity	Persistent excess	SD	97	Dopamin
**9**	Acromegaly	4	> 30	Yes	No	Extension into clivus and 3rd ventricle	Re-Inspection	Not reachable	Persistent excess	PD	51	Somatostatin
**10**	Acromegaly	2	10 < 20	No	No	Cavernous sinus	Re-Inspection	Risk of morbidity, venous bleeding	Persistent excess	PD	5	Repeat surgery @ 5 m, somatostatin
**11**	Acromegaly	4	10 < 20	Yes	No	Cavernous sinus	Re-Inspection	Risk of morbidity, venous bleeding	Persistent excess	SD	8	Somatostatin
**12**	Prolactinoma	4	20 < 30	Yes	No	Suprasellar extension to optic chiasm	Re-Inspection	Risk of morbidity, max safe resection achieved	NA	NA	NA	Lost to follow-up
**13**	M. Cushing	4	10 < 20	Yes	Yes	Intra- and suprasellar, dense tumor	Additional Resection	Inadvertent remnants after surgery	Persistent excess	SD	23	Somatostatin + radiation therapy
**14**	M. Cushing	4	20 < 30	Yes	No	Extension into clivus	Additional Resection	Risk of morbidity	Remission	SD	14	
**15**	Acromegaly	2	10 < 20	No	No	Covering ICA	Additional Resection	2^nd^ iMRI showed maximum safe resection	Persistent excess	SD	4	NA
**16**	Prolactinoma	1	20 < 30	Yes	No	Suprasellar extension to optic chiasm	Additional Resection	Risk of morbidity	Persistent excess	SD	82	Watch-and-wait
**17**	Acromegaly	4	20 < 30	Yes	No	Suprasellar, cavernous sinus	Additional Resection	Venous bleeding, residual not reachable	Persistent excess	NA	4	Transcranial surgery

### Clinical outcome

Hormonal remission could be achieved in 67 cases in our cohort (59%), whereas 36 (32%) exhibited persistent hormone excess at last follow-up. No endocrinologic follow-up was available for 11 cases (10%) (Table 2).

In 36 cases with persistent hormone excess after surgery, 18 (50%) received adjuvant medical therapy, 3 (8%) were treated by additional radiation therapy, and 3 (8%) had subsequent surgery. For medical therapy, dopamine analogues (*n* = 9) and somatostatin (*n* = 9) were applied. Close surveillance was chosen for all other cases.

The rate of persistent hormone excess was significantly higher after incomplete resection of FPAs (13/19 cases, 68%) compared to cases with a complete tumor removal (23/84 cases, 27%) (*p* = 0.001). In both cases with false-negative iMRI findings, additional medical therapy was administered due to persistent hormone oversecretion after surgery.

In cases of complete hormonal remission, we did not observe adenoma recurrence during follow-up MRI in our cohort. Three recurrences with progressive tumor growth were observed in incompletely resected FPAs with persistent hormone excess after a median of 26 months after initial surgery (range 6–51 months).

### Variables of persistent hormone excess after surgery

Risk factors for persistent hormone excess after surgery were evaluated by regression analysis (Table 5). Incomplete tumor resection strongly indicated a higher risk for failure to control hormone levels after transsphenoidal resection (OR 7.13, *p* = 0.002) while younger age indicated a more favorable outcome with regard to hormonal remission (OR 0.96, *p* = 0.01). There was a trend towards a better control of hormone excess in Cushing’s disease compared to other entities in this series (OR 0.34, 95% C.I. 0.11–1.10, *p* = 0.07).

**Table 5:** Univariate regression for variables of persistent hormone excess

	HR	95% C.I		*p*-value
		Lower	Upper	
Sex (f/m)	1.46	0.63	3.37	0.37
**Age** (cont.)	**0.96**	0.93	0.99	**0.01**
Visual deficits (y/n)	1.04	0.32	3.37	0.95
M. Cushing (y/n)	0.34	0.11	1.10	0.07
Acromegaly (y/n)	1.64	0.72	3.70	0.24
Prolactinoma (y/n)	1.18	0.49	2.80	0.72
Previous pituitary surgery (y/n)	2.99	0.88	10.23	0.08
Previous medical therapy (y/n)	1.18	0.49	2.80	0.72
Knosp grade (cont.)	1.32	0.96	1.82	0.09
Sagittal size (cont.)	1.60	0.95	2.69	0.08
Contact with optic chiasm (y/n)	0.79	0.34	1.84	0.58
Only microscopic surgery (y/n)	1.63	0.69	3.85	0.26
Use of navigation (y/n)	0.93	0.41	2.10	0.86
**Incomplete tumor resection** (y/n)	**7.13**	2.05	24.76	**0.002**

**Bold** indicating significance, *HR*: hazard ratio

As indicated in Table 4, normalization of hormonal overexcretion was an uncommon observation when residual tumor was left behind after iMRI in 4/17 cases (24%) with one of these cases having an immature follow-up of 2 months precluding final conclusions on this issue.

### Complications

Overall, postoperative complications were rare. Among all 114 cases, we did not observe postoperative intracranial hemorrhage or surgery-related mortality. Progressive visual impairment was observed in one case, however not yielding any conclusive findings within the cavity during revision surgery. One case was suspicious for postoperative meningitis, which was successfully treated by administration of antibiotics. Seven cases (4.8%) had rhinoliquorrhea which was treated by lumbar drainage and lead to revision surgery in one case (0.8%).

Postoperative diabetes insipidus needing treatment by titrated doses of desmopressin during the postsurgical period occurred in 9 cases (8%). One patient (0.8%) required permanent substitution therapy. Transient postoperative hyponatremia was observed in 7 cases (6%) which resolved without specific treatment during hospital stay. Median hospital stay was 8 days (range 5–21 days).

Upon follow-up 8–12 weeks after surgery, 25 cases (17%) showed signs of new pituitary insufficiency. The adrenocorticotrope axis was affected in the majority of cases with subsequent requirement of cortisol substitution therapy (*n* = 22). Eleven patients required new thyrotrope hormone substitution and 4 patients required new gonadotrope substitution therapy, respectively.

We screened for risk factors for the development of new postoperative pituitary insufficiency using regression analysis, but did not detect significant variables in our data. Particularly, re-inspection of the resection cavity (OR 1.40, 95% C.I. 0.56–3.50, *p* = 0.47), additional tumor removal after iMRI (OR 1.63, 95% C.I. 0.58–4.62, *p* = 0.36), and a complete tumor removal (OR 2.43, 95% C.I. 0.76–7.79, *p* = 0.14) were not associated with observation of new pituitary insufficiencies. This underscores the safety of iMRI- guided surgery with no signs for additional morbidity attributable to the procedure.

## Discussion

In this study, we sought to evaluate the role of iMRI in TSS for FPAs with active hormone secretion in a consecutive retrospective cohort. While iMRI was suggestive for residual tumor in 45% of FPA cases (51/114), less than half of those cases were eventually amenable to further tumor resection (22/114, 19%). Re-inspection of the resection cavity ruled out equivocal iMRI findings without detection of residual tumor in 20/114 cases (18%) and resections remained incomplete despite iMRI in 19/114 cases (17%). Complete resections correlated strongly with hormonal remission during follow-up and corroborated the impact of surgical treatment in FPAs.

### Intraoperative MRI in FPA resections

While iMRI is a well-established tool in glioma surgery, its use in pituitary surgery is also increasing. Numerous reports in the literature have highlighted the benefits of this technique, supporting high rates of complete adenoma resections in microscopic as well as endoscopic surgery. However, the need for additional resections after iMRI was highly variable at rates of up to 50–82%, according to recent studies and meta-analysis[18–20]. In endoscopic procedures, it is hypothesized that additional resections could be required less frequently due to better intraoperative visualization[20–22]. However, FPAs are often underrepresented in existent studies limiting the share of experience in these cases with high potential benefit from complete resection and intraoperative confirmation of extent of resection[23].

Our study presented a different role of iMRI in sole FPAs with active hormone secretion compared to previous series, however. In our series, additional tumor was removed in only 19% of cases and in another 14%, re-inspection cleared equivocal findings without detection of residual tumor. As a bottom line, surgical results were influenced by iMRI in 33% of cases in our study while outcome was unlikely affected by iMRI in the remaining 2/3. Moreover, we observed tumor size and invasiveness being persistent boundaries to the completeness of a resection despite use of iMRI. While the influence of iMRI on surgical results in 33% of FPAs is not insignificant, it has to be argued whether it is worth the extra time and expense needed for iMRI-guided procedures based on this data.

Despite this finding, studies report high sensitivity and specificity for iMRI detection of residual tumor in non-functional adenomas proclaiming a convincing efficacy in guiding transsphenoidal surgery[20, 24]. From our clinical experience, FPAs require greater diligence when it comes to delineating remnants on iMRI at 1.5 T. This is reflected by a moderate 68.6% positive predictive value of iMRI findings in our study, since 16 suspected tumor remnants proved false-positive upon further exploration. Thus, the ability for re-inspection of the resection cavity to clear equivocal findings on iMRI reflects a key element of iMRI-guided pituitary surgery in order to prevent unanticipated remnants and to confirm complete resections as accurately as possible. When tumor remnants were excluded on iMRI however, high negative predictive value (96.8%) implies high diagnostic quality of negative iMRI findings, which could repeal the need for additional postoperative MRI.

Nevertheless, the clinical significance of rare false-negative MRI findings has to be considered, as both respective cases in our series required further medical treatment due to persistent hormone oversecretion.

Higher imaging accuracy is discussed for MRI scanners at 3 T, which could inherit advantages especially with regard to intraoperative tumor delineation in confined spaces like the sellar region. However, the benefit of higher field strength and better resolution has to be weighed against the greater susceptibility for image artifacts in the intraoperative environment [10, 25, 26].

What we can derive from our series is that FPAs exhibit a more ambiguous appearance on iMRI compared to non-functioning adenomas or gliomas. This makes delineation more difficult and requires careful analysis including focused re-inspections of the resection cavity in order to pursue optimal surgical results. On an individual basis, iMRI is a valuable and reliable tool to detect intraoperative tumor remnants in FPAs but the balance of efforts and gains limit a generous recommendation for its application so far.

### Implications on EOR

Previous reports supported the use of iMRI to enhance complete resections which also affects hormonal remission in FPAs[1–4, 20]. Studies have identified anatomical limitations to the EOR in TSS generally in terms of tumor size (macroadenoma), suprasellar extension to the optic system, and parasellar invasion into the cavernous sinus (e.g., Knosp grade)[19, 21, 27]. As a consequence, gross tumor removal is sought possible in only 2/3 of cases based upon evaluation of preoperative MRI albeit final resection results often exceed initial expectations with complete resection rates of 80–90% in larger series of predominantly non-functioning adenomas[6, 23, 28].

Our results were in line with these data. In a first attempt, a complete resection was observed in 55% on iMRI and further inspection and resection eventually enabled gross adenoma removal in 83%. Adenoma size and invasiveness were significant risk factors for incomplete resections despite iMRI, while FPA subtypes did not impact on EOR in our series.

We found that neither previous surgical nor medical treatment had an impact on EOR, which could point out a benefit of iMRI-guided surgery allowing to overcome some of the challenges regarding identification of microadenomas or delineation of scarred and altered tissues encountered during FPA surgery [3, 11, 13].

### Impact on hormonal remission rates

Surgical treatment of FPAs does not only aim at decompression of nervous structures but also convey the prospect of hormonal remission. According to the literature, remission can be achieved in 50–90% of surgical cases strongly depending on the completeness of surgical adenoma removal but also on the type of adenoma. Particularly, tumors with parasellar invasion and macroadenomas are associated with reduced rates of hormonal remission, which corresponds to the greater risk for incomplete resections associated with these parameters[2–4, 13, 14, 17]. Consistent with these findings, incomplete resection was the single most significant driver of persistent hormone excess increasing the risk by sevenfold in our series (Table 5).

Aside from the ambiguous value of iMRI based on observed rates of additional resections or focused re-inspections in our study, the strong correlation of EOR and hormonal outcome in turn underscores the importance and the beneficial prognostic impact of a complete adenoma removal which may warrant the efforts taken to optimize EOR by iMRI, nevertheless.

Inclusion of medically pre-treated prolactinomas into our cohort limits the generalizability of our results regarding the effect of surgery on hormonal outcome and might contribute to remission rates appearing at the lower end of the expected range in this study. However, our primary aim was to evaluate iMRI for transsphenoidal surgery in challenging conditions like FPAs, which accounted for this approach for inclusion.

For treatment of Cushing’s disease, our data suggests a better ability to achieve hormonal remission after surgery (Table 5). However, taking into account the high frequency of microadenomas (70%) combined with the low number of incomplete resections (*n* = 3) in this entity, this observation is potentially biased by included tumor sizes and a limited sample size in this subgroup preventing to draw balanced conclusions.

### Impact of surgery on pituitary function

As a general risk in pituitary surgery, new pituitary insufficiency can occur after surgery with reported rates of up to 60% in some series[29, 30]. Even though these high rates are partially be explained by heterogeneous definition and follow-up periods, usually new hormone insufficiencies are observed in about 11–22% of cases[19, 31, 32]. Overall, our 17% rate of new insufficiencies found at a median of 8 months after surgery was consistent with these findings. Since recovery of hormone function is expected to occur 6–18 months after surgery, our follow-up data might have been immature in order to draw final conclusions regarding the insufficiency rate[33, 34].

While the literature does not provide sustainable risk factors for surgery-related pituitary insufficiency other than general surgical invasiveness, the rate of permanent diabetes insipidus of 0.8% in our series is considerably lower than previously reported[19, 31, 32, 35]. In our series, neither a focused re-inspection, nor additional adenoma resection after iMRI, nor the achievement of GTR was a significant risk factor for new hormone insufficiency after surgery. In our opinion, this underscores the safety of iMRI-guided procedures which hypothetically result in a less invasive surgical approach. Contributing to the argument about the value of iMRI, the absence of significant medical drawbacks at least provides data to optionally apply this technique in FPA surgery on an individual basis.

### Limitations

This consecutive series of FPA resections exhibits typical limitations originating from its retrospective nature. Essentially, heterogeneity of data regarding tumor sizes, inclusion of revision surgeries, infiltrative growth behavior of FPA, and variation in follow-up periods for imaging and hormone examinations limits our conclusions and the generalizability of our results to all pituitary adenomas.

Thorough analysis of postoperative MRI and endocrine follow-up according to consensus criteria reflects our effort to define robust outcome endpoints. Nevertheless, we lost 11 cases (10%) for follow-up of hormonal outcome status after surgery.

With all patients treated surgically with iMRI guidance in this series, no conclusions for the effect of iMRI per se can be drawn from our data. However, our science of practice stressed the importance of a complete tumor resection in FPA which grants support to our approach of routinely combining iMRI with conventional microscopic surgery.

EOR was not evaluated volumetrically in our series which left analysis of surgical outcome to a binary variable. While volumetry is rigorously performed in glioma research nowadays, we focused on invasion or entanglement of para- and suprasellar structures in our analysis. In our opinion, such conditions impose greater limitations on surgical outcome in pituitary surgery compared to volumetric size.

## Summary and conclusions

This large retrospective series of functional pituitary adenomas underscores the strong impact of complete resections to control excess hormone secretion regardless of its entity. This supports the use of intraoperative imaging to achieve a complete resection whenever safely possible with iMRI being a versatile adjunct to transsphenoidal resections in this regard. Tumor remnants could reliably be detected in large and invasive tumors and equivocal iMRI findings could be cleared in a subsequent focused re-inspection to rule out tumor remnants intraoperatively. In conclusion, efforts taken and benefits gained by an iMRI-guided approach should be carefully balanced on an individual basis. The use of iMRI may allow for a less invasive surgical approach than usually required in FPA possibly contributing to low complication rates.

## Data Availability

The data that support the findings of this study are available on request from the corresponding author (M.S.). The data are not publicly available due to contained information compromising privacy necessitating informed consent.
